# Changes in a sensorimotor network, occipital network, and psychomotor speed within three months after focal surgical injury in pediatric patients with intracranial space-occupying lesions

**DOI:** 10.1186/s12887-022-03348-5

**Published:** 2022-06-01

**Authors:** Xue-Yi Guan, Wen-Jian Zheng, Kai-Yu Fan, Xu Han, Xiang Li, Zi-Han Yan, Zheng Lu, Jian Gong

**Affiliations:** Department of Pediatric Neurosurgery, Beijing Tiantan Hospital, Capital Medical University, Beijing, (100070) China

**Keywords:** Brain network, Pediatric, Brain surgery, Psychotomotor speed, Intracranial space-occupying lesions, Injury

## Abstract

**Background:**

Studies on cognition and brain networks after various forms of brain injury mainly involve traumatic brain injury, neurological disease, tumours, and mental disease. There are few related studies on surgical injury and even fewer pediatric studies. This study aimed to preliminarily explore the cognitive and brain network changes in children with focal, unilateral, well-bounded intracranial space-occupying lesions (ISOLs) in the short term period after surgery.

**Methods:**

We enrolled 15 patients (6–14 years old) with ISOLs admitted to the Department of Pediatric Neurosurgery of the Beijing Tiantan Hospital between July 2020 and August 2021. Cognitive assessment and resting-state functional magnetic resonance imaging (rs-fMRI) were performed. Regional homogeneity (Reho), seed-based analysis (SBA) and graph theory analysis (GTA) were performed. Paired T-test was used for statistical analysis of cognitive assessment and rs-fMRI. Gaussian random-field theory correction (voxel *p*-value < 0.001, cluster *p*-value < 0.05) was used for Reho and SBA. False discovery rate correction (corrected *p* value < 0.05) for GTA.

**Results:**

Our results showed that psychomotor speed decreased within three months after surgery. Further, rs-fMRI data analysis suggested that sensorimotor and occipital network activation decreased with low information transmission efficiency.

**Conclusion:**

We prudently concluded that the changes in cognitive function and brain network within three months after surgery may be similar to ageing and that the brain is vulnerable during this period.

**Supplementary Information:**

The online version contains supplementary material available at 10.1186/s12887-022-03348-5.

## Background

Pediatric brain tumours are the most common solid tumours in children [1]. The prognosis of many childhood brain tumours has improved steadily in recent decades [2]. Currently, the average 5-year survival rate is close to 75%. For low-grade gliomas, such as pilocytic astrocytoma, the average 20-year survival rate is close to 90% [3, 4]. At present, surgery is still the primary treatment for intracranial space-occupying lesions (ISOLs), such as brain tumours, focal brain degeneration, etc. Neurosurgical operations can cause damage to the brain tissue while treating the disease. One of these manifestations is cognitive impairment [5].

Resting state fuctional magnetic resonance imaging (rs-fMRI) is an essential tool in neuroscience because it can non-invasively reveal the activities inside the brain and construct a functional network of the resting state. It can show the mechanism of the brain network level behind cognitive impairment and neurological deficits [6].

Many studies have used rs-fMRI in clinical practice. It involves a variety of fields, such as mental and psychological diseases, neurodegenerative diseases, cerebral tumours, and even changes in the brain network in Crohn’s disease [7–10]. At present, rs-fMRI studies on human brain injury mainly focus on brain network changes in patients with stroke, traumatic brain injury, and brain tumours after multidisciplinary treatments [11–13]. However, rs-fMRI studies on injuries caused by surgery alone are rare, especially in the pediatric population. Our study aimed to investigate changes in the brain network in children with low-grade or non-neoplastic ISOLs before and after surgery. Patients with low-grade or non-neoplastic ISOLs do not require adjuvant treatment after surgery. Therefore, the effect of the surgery itself on the brain network and cognition can be well studied. According to our observations, the cognitive impairment in children after surgery-only treatment is not statistically evident, and our study may provide some hints for the principle behind the phenomenon.

## Methods

### Participants

Twenty ISOL patients who were admitted to the Department of Pediatric Neurosurgery of Beijing Tiantan Hospital from July 2020 to August 2021 were included. Preoperative cognitive assessment and rs-fMRI scan were performed. The same flow would perform again within three months after surgery.

**Inclusion Criteria:** (a) Age: 6–16 years; (b) Disease: Primary ISOLs; (c) Brain surgery is required.

**Exclusion Criteria:** (a) Children or their parents did not agree to participate in our study; (b) Children were diagnoised with hydrocephalus (Evan index > 0.3); (c) The children are unable to finish the rs-fMRI scan for any reasons; (d) The quality of imaging data is low, such as excessive head movement or awful normalization; (e) The patients had a history of craniocerebral trauma, craniocerebral surgery, mental and psychological diseases, genetic metabolic diseases, endocrine system diseases, etc.; (f) Pathological grade is above WHO 3; (g) The children have contraindications of general anesthesia; (h) The children are unable to follow-up.

Poor normalization was found in one child during data preprocessing, and the other four children were excluded due to excessive head movement during scanning. Finally, a total of 15 children were enrolled.

### Imaging data acquisition

The imaging protocol included a three-dimensional T1-weighted (3D-T1) imaging sequence and an echo-planar imaging (EPI) sequence sensitive to blood oxygenation level dependent (BOLD) signal. All imaging data were obtained using the Siemens Prisma 3.0 Tesla MR system in Beijing Tiantan Hospital. A standard 64-channel head coil was used for signal reception. Every child lay supine with the head snugly secured by a belt and foam pads.

The parameter information of 3D-T1 series is: repetition time (TR) = 1560 ms, echo time (TE) = 1.65 ms, acquisition matrix = 256 × 256 × 176, slice thickness = 1 mm, flip angle = 8.

In EPI scans, children were instructed to close their eyes but remain asleep,. The scanning parameters were as follows: TR = 2000 ms; TE = 35 ms; voxel size = 2.2 × 2.2 × 2.2 mm; field-of-view (FOV) = 256 mm; slice thickness = 2.2 mm; the number of slices = 69; and total scanning time is 8 min and 240 volumes were acquired.

### Preprocessing data

Data Processing and Analysis of Brain Imaging (DPABI v6.0,http://rfmri.org/dpabi) and Statistical Parametric Mapping (SPM8, http://www.fli.ion.ucl.ac.uk/spm), implemented in MATLAB (Matlab Release 2020b, Mathworks Inc., Natick, MA), were used to preprocess rs-fMRI images [14, 15]. Before preprocessing, the mask of every individual lesion was drawn and deleted from the corresponding individual 3D-T1 image using MRIcron by an experienced neurosurgeon for better normalization [16, 17]. The lesion masks were checked by another experienced neurosurgeon. The preprocessing steps were as follows: (a) Discard the first 10 images; (b) Slice-timing correction and head motion correction. Exclude the subjects whose head movement translation is greater than 3 mm or rotation greater than 3°. Four children were excluded due to excessive head movement during the MRI scan. (c) Redirect the functional image to the standard space, Montreal Institute of Neurology space (MNI space), by DARTEL [18], and then resample to 3 mm cube; (d) The linear trend, cerebrospinal fluid signal, white matter, and Friston 24-parameter head motion model were regressed as cumulative covariates from BOLD signal [19]. Covariate regression of global signals was not used during preprocessing [20, 21]; (e) Smoothing the images with full width at half maximum 4 × 4 × 4; (f) Band-pass filtering (0.01 Hz-0.10 Hz).

### Brain network analysis

Rs-fMRI data analysis methods can be generally divided into two categories: functional separation method, such as regional hemogeneity (Reho) analysis, the amplitude of low-frequency fluctuations (ALFF), fractional ALFF, etc. and functional integration method for example seed-based analysis (SBA), graph theory analysis (GTA), etc. [22].

We used the DPABI toolbox to calculate Reho, one of the methods of functional separation. For the functional integration method, we used DPABI to perform seed-based analysis with Dosenbach atlas, and DpabiNet to conduct global GTA (clustering coefficient, local efficiency, global efficiency, small-worldness, modularity, and characteristic shortest path length) [23]. The whole correlation method and Z transform were used to construct the network matrix. GTA used a weighted network, with the sparsity range using the default 0.1–0.34. Random times were set to 100 as default.

### Cognitive assessment

The chilidren’s cognitive functions were assessed using the CNS Vital Signs (CNS VS) battery [24]. The assessment was performed preoperatively and an rs-fMRI scan was performed within one week after the assessment. The same procedure would perform within three months postoperatively. We obtained cognitive data from 12 patients because of the other three’s poor cooperation.

### Statistical analysis and result display

For rs-fMRI imaging data that have converted to Z values by Fisher’s z transformation, paired T-test was performed with gaussian random-field theory (GRF) correction (voxel p-value < 0.001, cluster p-value < 0.05) for Reho and SBA, with false discovery rate (FDR) correction (corrected p value < 0.05), DpabiNet default option, for GTA. The cluster whose size is over 30 voxels are considered as an effective cluster [25]. Cognitive test data were performed paired T-test by SPSS (IBM SPSS Statistics version 24, IBM Corporation). The results were displayed by BrainNet Viewer [26].

## Results

### Clinical characteristics of pediatric patients

**Fig. 1:**
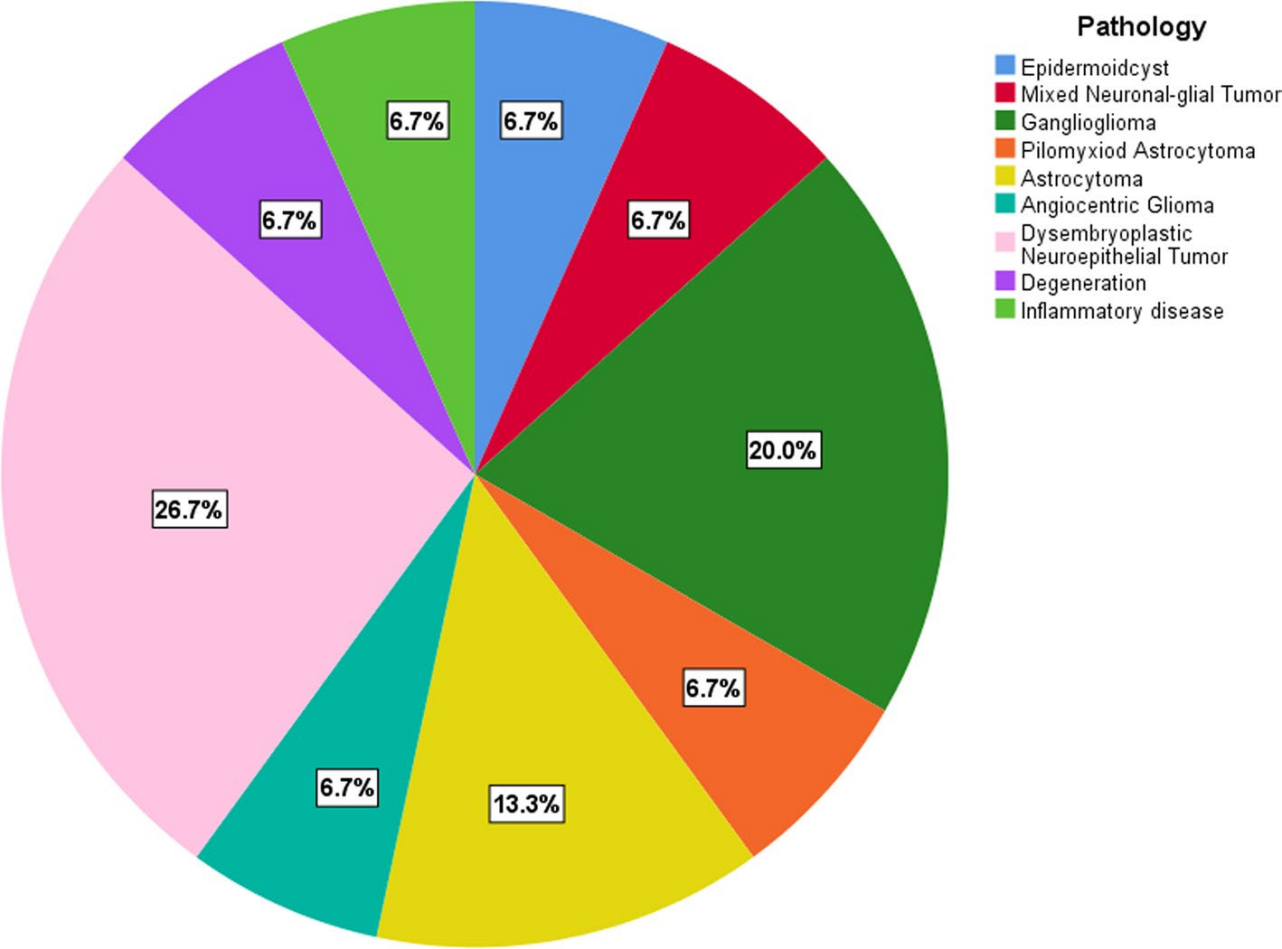
Pathological results of 15 enrolled patients. The pathological results of the 15 enrolled patients are ISOLs

Twenty patients were supposed to be enrolled but five patients were excluded. One for poor normalization and four for excessive head motion. Finally, 15 patients were enrolled. The clinical characteristics of the patients have been listed in Table 1. The mean age of patients was 10.40 (± 2.60) years, ranging from 6 to 14 years. The number of male is nine (9/15). The onset symptoms were mainly epileptic seizure (12 cases), followed by headache (two cases). There was one case asymptomatic. Five cases (5/10) had taken antiepileptic drugs before operation. Two of them received levetiracetam and three received valproate. Among the 15 enrolled patients, two cases were non-neoplastic lesions (one was degeneration of brain tissue, the other was inflammatory lesions). The other 13 cases were neoplastic lesions. The details of pathological results of enrolled patients were shown in Fig. 1. Ten (10/15) had lesions on the left side. The specific locations of the lesions were shown in Table 1. The mean follow-up interval was 79.20 (± 51.49) days, ranging from 5 to 154 days. Lesion volume could be approximately calculatedusing the formula for spheroid: V = 4/3π × a/2 × b/2 × c/2 (a and b = maximum perpendicular diameters on the axial images; c = diameter in the coronal direction on the sagittal or coronal images) [27, 28]. The mean volume was 6661.90 (± 7392.71) mm^3^, with a minimum of 153.90 mm^3^ and a maximum of 23,487.20 mm^3^. All lesions were unilateral, focal, well bounded and did not cross the brain lobes. The surgical damage was confined to the lesion and its periphery. The damage is focal and minimal. All patients were uneventful after the surgery. All patients were instructed to have levetiracetam twice a day after surgery based on body weight.

**Table 1:** Clinical characteristics of pediatric patients

	Value/Ratio	Percentage
Age(mean ± SD)(years)^*^	10.40 ± 2.60	
Gender(male:female)	9:6	60.00%:40.00%
Initial Symptom (Seizure:Headache: Asymptomatic)	12:2:1	80.00%:13.33%:6.67%
Antiepileptic drugs (Taken:Not taken)^#^	5:10	33.33%:66.67%
Disease (Tumor:Not Tumor)	13:2	86.67%:13.33%
Lesion Location and Lateralization (Left:Right)	10:5	66.67%:33.33%
Location of lesions		
Frontal (Left: right)	9 (5:4)	60.00%
Parietal (Left: right)	2 (2:0)	13.33%
Temporal (Left: right)	3 (2:1)	20.00%
Occipital (Left: right)	1 (1:0)	6.67%
Lesion size(mean ± SD)(mm^3^)^!^	6661.90 ± 7392.71	
Degree of excision (Gross total:Subtotal)	13:2	86.67%:13.33%
Follow-up interval(mean ± SD)(days)^$^	79.20 ± 51.49	

^*^ Ranging from 6 to 14 years

^#^ Two patients levetiracetam and three valproate

!The minimum of 153.90 mm^3^ and the maximum of 23,487.20 mm^3^

^$^ From the first postoperative day to the follow-up day, ranging from 5 to 154 days

### Brain network analysis

The AAL3 atlas was used to label statistically significant areas in the result reporting. All coordinates belong to MNI space.

#### Reho changes

**Fig. 2:**
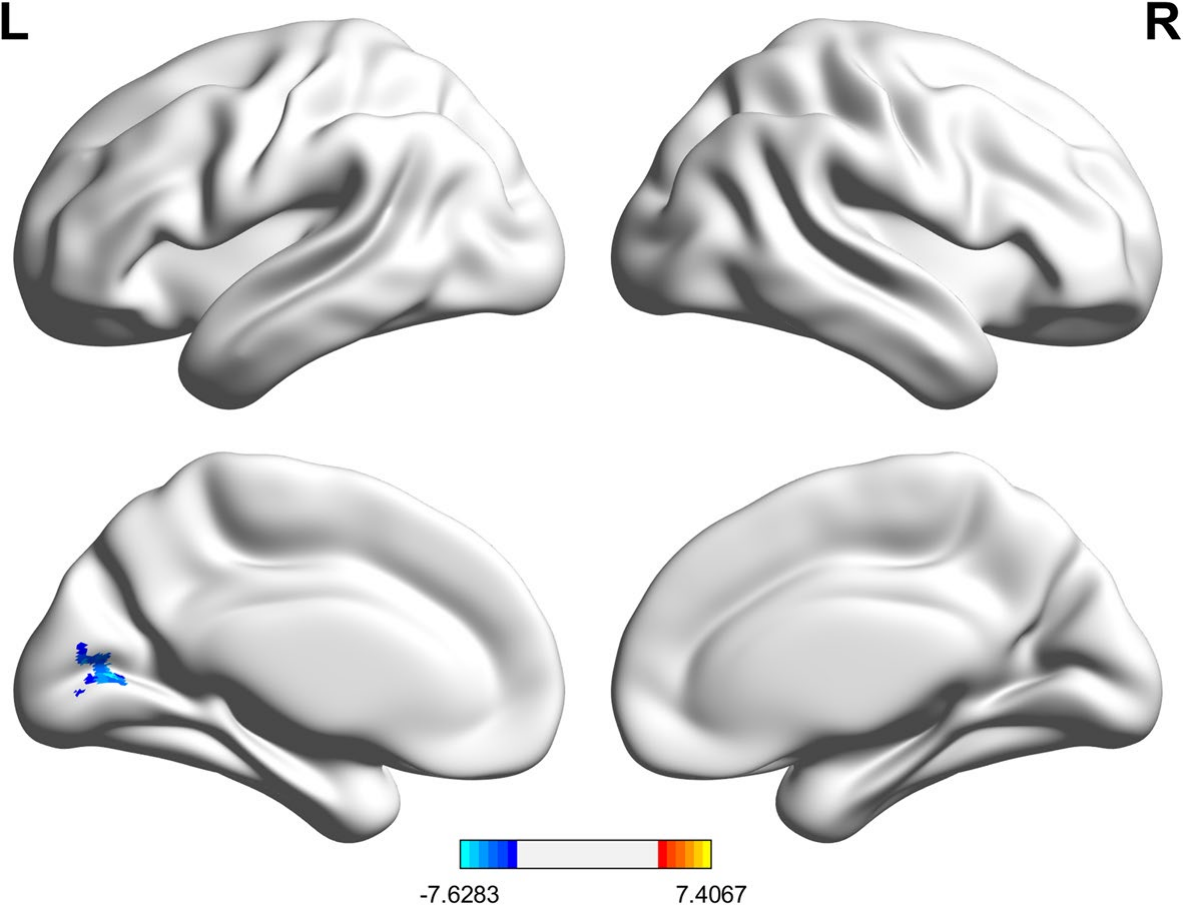
Reho changes. Postoperative Reho decreased significantly, with peak coordinates of (-9, -72, 9), and the anatomical location based on AAL3 atlas was left calcarine, belonging to occipital network of Dosenbach

The post-operation showed one Reho cluster (38 voxels, peak MNI (-9, -72, 9)) significantly lower than the pre-operation (t =—7.63). In the AAL3 atlas, the cluster covered left calcarine, left cuneus, and left lingual (Fig. 2). Detailed information regarding brain areas covered by the cluster is in Table 2.

**Table 2:** Reho changes

	Labels	Voxel number and percentage n (%)	Peak label
AAL3	Calcarine(L)	36(94.70%)	Calcarine_L
	Cuneus(L)	1(2.60%)	
	Lingual(L)	1(2.60%)	

*L* left

#### SBA

**Fig. 3:**
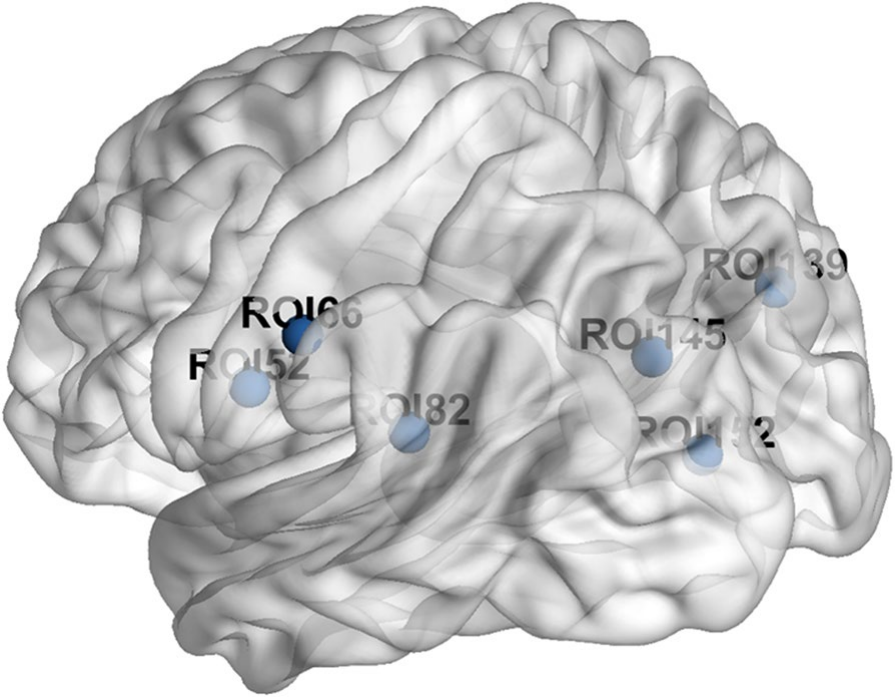
The seed points decreased significantly after surgery

**Fig. 4:**
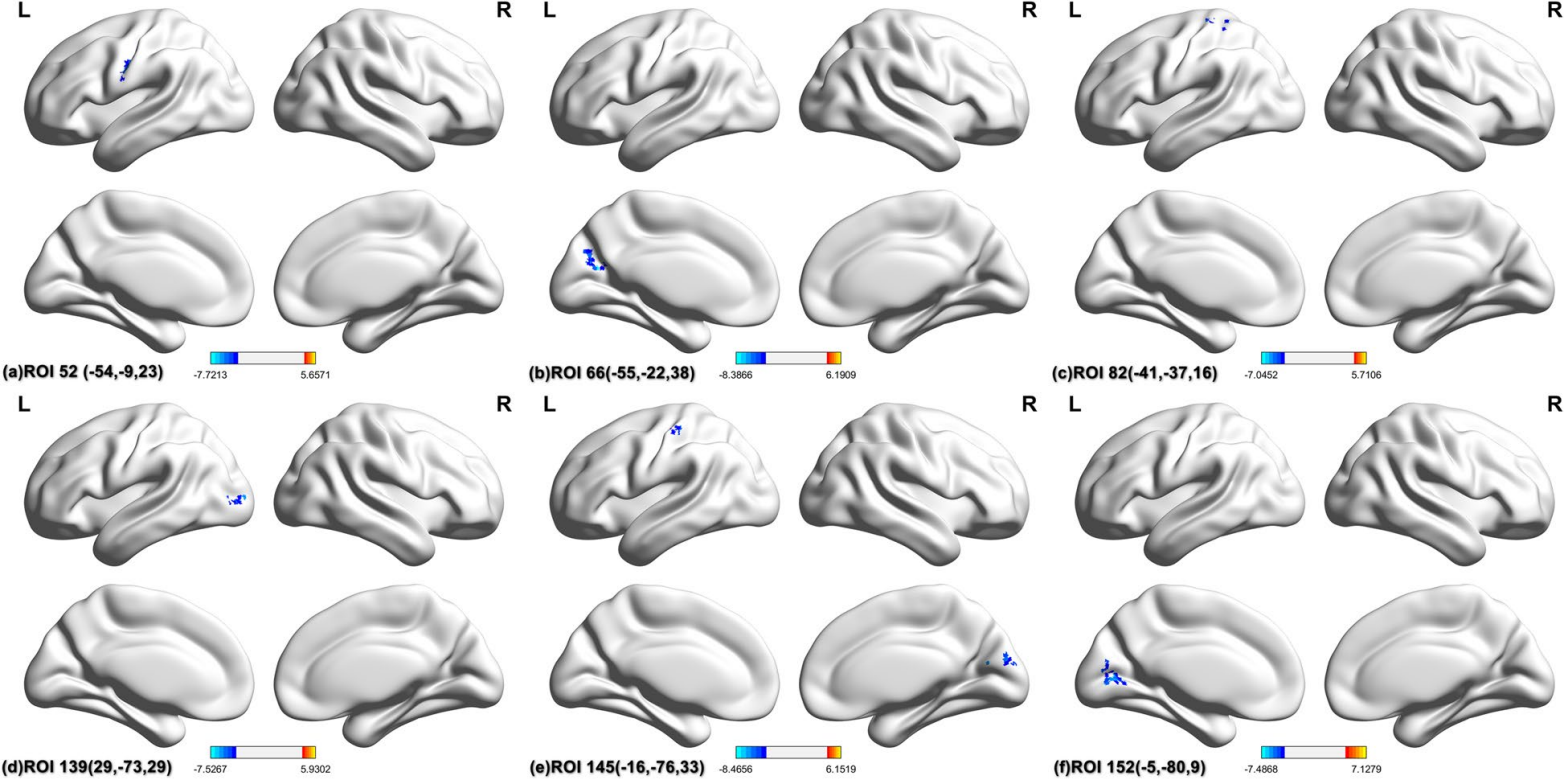
SBA results. The SBA results. (a)-(f) reveals the SBA results of the six seeds. After SBA on 160 seed points of Dosenbach atlas, six seed points were detected decreased significantly after surgery. The serial number and MNI coordinates of seed points have been marked in the figure. The anatomical position of each seed based on AAL3 is shown in Table 2. All of the seeds in the figure above and the regions of SBA results belong to sensorimotor network and occipital network of Dosenbach

We used Dosenbach atlas as seed points to calculate the functional connectivity between each seed point and the whole brain and compared the differences of every seed to whole-brain connectivity between pre-operation and post-operation. We calculated a total of 160 regions of interest (ROIs) and finally found that ROI 52 (-54, -9, 23), ROI 66 (-55, -22, 38), ROI 82 (-41, -37, 16), ROI 139 (29, -73, 29), ROI 145 (-16, -76, 33) and ROI 152 (-5, -80, 9), for a total of six seeds showed significant differences (Fig. 3 and Fig. 4). The details about peak t value, peak coordinates, voxel size, anatomical label, etc. are given in Table 3. We found that the six seeds all belong to the sensorimotor and occipital network of Dosenbach. We figured that these statistically significant decreased brain regions connected with these seeds are also roughly distributed in the sensorimotor network and occipital network according to their anatomical locations. We speculated that maybe the sensorimotor network and occipital network were injured. Therefore, we conducted the followed GTA.

**Table 3:** Seed-based analysis significant changes

Seeds location	Peak cluster location (AAL3)	Most overlapped areas (AAL3)	Peak coordinates (MNI space)	Peak t value	Total Voxel number
ROI 52 (-54,-9,23) ( Postcentral_L)	Postcentral (L) (41 vox)	Postcentral (L) (41 vox)	(-60, -6, 33)	-7.72	46
ROI 66 (-55,-22,38) (Parietal_Inf_L)	Calcarine (L) (10 vox)	Cuneus (L) (23 vox)	(-15, -66, 18)	-8.39	41
ROI 82 (-41,-37,16) (Temporal_Sup_L)	Postcentral (L) (23 vox)	Postcentral (L) (23 vox)	(-30, -45, 66)	-6.46	37
ROI 139 (29,-73,29) (Occipital_Mid_R)	Occipital Mid (L) (30 vox)	Occipital Mid (L) (30 vox)	(-36, -81, 6)	-7.16	30
ROI 145 (-16,-76,33) (Cuneus_L)	Cuneus (R) (25 vox)	Cuneus (R) (25 vox)	(12, -84, 21)	-8.47	36
ROI 145 (-16,-76,33)^#^ (Cuneus_L)	Postcentral (L) (32 vox)	Postcentral (L) (32 vox)	(-51, 24, 57)	-6.29	34
ROI 152 (-5,-80,9) (Calcarine_L)	Calcarine (L) (33 vox)	Calcarine (L) (33 vox)	(-18, -69, 6)	-6.82	41

^#^ ROI 145 has two clusters significant

*L* left, *R* right, *Sup* superior, *Inf* inferior, *Mid* middle

#### GTA

Nodes of the Dosenbach sensorimotor network and occipital network were extracted for GTA, and only global indicators were analyzed. We found that local efficiency (t = -2.69, *p* = 0.02), global efficiency (t = -2.70, *p* = 0.02) and characteristic shortest path length (t = 2.51, p = 0.03) are statistically significant differences. We speculated that the three topological properties indicated that the information transfer efficiency of the two networks was damaged.

### Cognitive assessment scores

The CNS VS battery is a complex assessment battery with various cognitive domain, which is a computer administered neuropsychological assessment tool [24]. The battery can provide a subject with a quick (30–40 min) evaluation and generate a report with age-adjusted standard scores for 15 domains, which are derived by 10 subtests. The 15 domains include Composite memory, Verbal memory, Visual Memory, Psychomotor Speed, and Reaction Time, Complex Attention, Cognitive Flexibility, Processing Speed, and Executive Function, Social acuity, Reasoning, Working memory, and Sustained attention, Simple Attention, and Motor Speed. According to all the 15 scores, a neurocognition index (NCI), a general assessments of the overall neurocognitive status of the patient, will be generated. The CNS VS standard scores have a mean of 100 and a standard deviation of 15. We focused on the 16 scores because they correspond to the clinical domain and are better understood. Based on our data from 12 patients, we found no statistical difference between preoperative and postoperative scores in these 16 domain scores (Table 4).

**Table 4:** Cognitive assessment scores comparison

Clinical Domain	mean	SD	T value	*P* value
NCI	-0.82	19.34	-0.14	0.89
Composite Memory	7.82	26.11	0.99	0.34
Verbal Memory	15.27	31.91	1.59	0.14
Visual Memory	-1.27	16.92	-0.25	0.81
Psychomotor Speed	-5.64	10.02	-1.87	0.09
Reaction Time	-9.82	21.77	-1.50	0.17
Complex Attention	-1.46	30.49	-0.16	0.88
Cognitive Flexibility	4.73	26.87	0.58	0.57
Processing Speed	-4.73	16.08	-0.98	0.35
Executive Function	3.00	26.68	0.37	0.72
Simple Attention	8.82	22.26	1.31	0.22
Motor Speed	2.09	15.17	0.46	0.66
Social Acuity	-2.55	11.56	-0.73	0.48
Reasoning	0.91	8.98	0.34	0.74
Sustained Attention	-25.73	73.66	-1.16	0.27
Working Memory	1.18	21.18	0.19	0.86

## Discussion

Research on cognitive function and brain network changes after various forms of brain injury is primarily focused on traumatic brain injury (TBI), stroke, neurological diseases, tumour effects, and mental diseases [29–31]; and the participants are most adults. Few studies have focused on the impact of surgery-only on brain networks and cognitive function. Additionally, there have been few studies on children. Our study preliminarily focused on cognitive function and brain network changes in children after brain surgery in short term period.

All enrolled patients were children (6–14 years old). The surgical lesions were unilateral and focal and did not involve deep brain structures. Under these conditions, we found that all the 16 scores of the CNS VS battery did not reach the level of statistical significance. However, the p-value of psychomotor speed (*p* = 0.09) was the lowest among all the scores, which was closest to the level of statistical significance. The t-value of psychomotor speed (t = -1.87) decreased most among all the 16 scores. According to the results, we assumed that the patients' psychomotor speed was probably decreased after surgery, despite it was not statistically significant. For rs-fMRI, Reho and SBA indicated that activation of sensorimotor network and occipital network were decreased. The GTA implied the low efficiency of the two networks post-operatively.

The psychomotor speed of CNS VS is designed to measure how well a subject perceives, attends, responds to visual-perceptual information and performs motor speed and fine motor coordination. It is calculated based on the scores from the finger-tapping test and the symbol digit coding test. In the current study, children showed decreased psychomotor speed after surgery. In a long-term study of patients with temporal lobe epilepsy, researchers found substantial reductions in attention and psychomotor speed, and no significant changes in other domains of cognition compared with normal subjects [32]. This type of deterioration is mainly related to the duration of epilepsy, age of onset, history of tonic–clonic seizures, and low educational level. Frequent episodes could impair brain function, and this study found that one manifestation of this impairment is a decrease in psychomotor speed. Era et al. [33] performed a psychomotor speed test on a random sample of nearly 8,000 people aged 30 years and older. They concluded that the decline in psychomotor speed during ageing occurs at a very young age. This decline accelerates after the age of 70 years. The psychomotor speed may be an indicator of ageing. The study by Amieva et al. further proposed that psychomotor speed reduction is an indicator of normal ageing and is associated with an increased risk of various brain outcomes such as dementia, Alzheimer’s disease, Parkinson’s disease, disability, and depression [34]. According to Amieva et al., low psychomotor speed may not be specific to any neurocognitive syndrome. Nevertheless, it may be a common feature of various neurocognitive syndromes, making it a good candidate for assessing an individual’s brain vulnerability as they age [34]. Our findings of the cognitive test suggest that surgical damage may lead to a low psychomotor speed, similar to normal ageing. It further indicates that psychomotor speed could reflect the brain’s vulnerability to some extent. Our rs-fMRI results showed that, the sensorimotor and occipital network intrinsic deactivation, and low efficiency could account for the low psychomotor speed after surgery.

For rs-fMRI results, the statistically significant brain regions of Reho and SBA included the left calcarine sulcus, left posterior central gyrus, left inferior parietal lobule, left and right cuneus, left superior temporal gyrus, and left middle occipital gyrus. In summary, these structures belong to the sensorimotor and occipital networks. Anatomically, it is located in the posterior part of the brain. In an animal model study of pediatric TBI, fMRI scans were performed on piglets surgically modulated into TBI models [35]. Compared to controls, the Pearson values and mean ratios of visual, executive control, and sensorimotor networks were significantly reduced in TBI piglets [35]. This finding is similar to our results and, to some extent, supports our imaging results. In their study, Cassady et al. compared the segregation of sensorimotor networks in young and old adults. They found that the sensorimotor networks in older adults were less segregated, and the older adults had worse sensorimotor performance than the young. Calculating network segregation aims to examine the within-network correlations in relation to between-network correlations. Network segregation is the ratio of the difference between the average connectivity within the network and the average connectivity between the networks to the average connectivity within the network [36]. Specifically, reduced network segregation means weak functional connections in the same functional network but strong functional connections between different networks. These findings link network segregation to neural specificity and suggest that segregation is a more sensitive predictor of age-related decline in sensorimotor behaviour. Network segregation was not calculated in our study. However, based on SBA results, some reduced internal connections of the sensorimotor and occipital networks and no significant change detected in connection with the whole brain, we could estimate that the separation of the whole of the two brain networks was reduced in postoperative children. Thus, the difference from Cassady et al.’s results may be the particularity of the brain network in children.

The occipital network of Dosenbach is located in the occipital lobe, which mainly involves visual processing. According to the results of our study, activation of the occipital network was decreased, but no apparent visual impairment occurred in any of the enrolled children after surgery. Changes in the optical network also existed in the study of the TBI model of piglets [35]. Perhaps our results can be better explained and reach similar conclusions to Cassady et al.’s study by considering both occipital and sensorimotor networks as networks of interaction with the outside world. In a study of interictal episodic migraineurs without aura, researchers concluded that the sensorimotor network was impaired and played a crucial role in pain regulation and chronicity. Their results also showed increased functional connectivity from the primary visual cortex to the sensorimotor network. Nonetheless, they cannot be explained by the syndrome well [37]. To the best of our knowledge, this phenomenon cannot be fully explained, and further research is required.

Barulli et al. reviewed the concepts of brain reserve (BR) and cognitive reserve (CR) [38]. BR refers to differences in brain size and other quantitative aspects (such as the number of synapses) that can explain different susceptibilities to functional impairment in the presence of pathological or other neurological impairments. Functional impairment occurs when an injury exceeds the BR threshold. CR refers to differences in cognitive processes and is the functional manifestation of the influence of lifelong intellectual activity and other environmental factors, which can explain the different susceptibilities to functional impairment in the presence of pathological or other neurological impairments. The CR model argues that cognitive processes are crucial to explaining the differences between functionally impaired and non-functionally impaired people, despite having the same brain changes or pathologies. These cognitive processes include differences in cognitive efficiency, ability, and flexibility, shaped by life experiences. Thus, CR is active in two ways: (a) it is more dependent than BR on current neural activity to account for functional differences, and (b) it suggests that current neural activity is shaped by different cognitive exposures/activities throughout life. CR is usually estimated using proxy variables of lifetime exposure and cognitive activity: years of education, measures of crystallized intelligence such as vocabulary or knowledge, literacy level, number of intellectually stimulating leisure activities, occupational complexity, and socioeconomic status are all commonly used to create estimates of the cognitive reserve [39]. As noted above, the cognitive assessment of patients in the current study is consistent with rs-fMRI results. Further, we cautiously suggest that the brain network level and cognitive level mechanisms of focal surgical injury may be similar to the normal ageing process. Combining the concepts of BR and CR, we assume that surgical resection of lesions consumes BR. Furthermore, children do not show statistically significant cognitive abnormalities, which may be the consumption of CR. Because CR is related to individual experience, children’s CR may not completely compensate, as shown in rs-fMRI. Therefore, allowing children to return to normal school life as soon as possible after surgery to promote the improvement of CR may encourage rehabilitation.

The current study has some limitations. Parents’ unwillingness to cooperate with follow-up makes obtaining more extended follow-up data challenging. Additionally, children’s poor self-control makes it challenging to minimise head movements in the long MRI scan, resulting in data loss. Further studies should re-do rs-fMRI scan and re-test the assessment to understand network changes after three months and expand the sample size to reduce the effects of noise.

## Conclusion

The psychomotor speed of children with unilateral, focal, and well-bounded ISOLs might be sensitive to brain damage within three months after surgery. The decreased activation and low efficiency in the sensorimotor and occipital networks support this theory. These findings imply that the brain is in a vulnerable period within three months after surgery. We cautiously suggest that the damage caused by this kind of surgery to the pediatric brain is similar to ageing.

## Fundings

This research was funded by the National Natural Science Foundation of China (No.81870834) and the Special project of peadiatrics of collaborative development centre of Beijing hospital administration (No. XTYB201817).

## Supplementary Information

**Additional file 1:** 

**Additional file 2:** 

## Data Availability

All data generated or analyzed during this study are included in the published article. Some or all data, models, or code generated or used during the study are available from the corresponding author by request.

## Abbreviations

3D-T1: Three-dimensional T1-weighted
ALFF: Amplitude of low-frequency fluctuations
BOLD: Blood oxygenation level dependent
BR: Brain reserve
CNS VS: CNS Vital Signs
CR: Cognitive reserve
EPI: Echo-planar imaging
FOV: Field-of-view
GTA: Graph theory analysis
ISOLs: Intracranial space-occupying lesions
MNI: Montreal Institute of Neurology
NCI: Neurocognition index
Reho: Regional homogeneity
ROIs: Regions of interest
rs-fMRI: Resting state fuctional magnetic resonance imaging
SBA: Seed-based analysis
TBI: Traumatic brain injury
TE: Echo time
TR: Repetition time
