# Supplementary material for: Changes in a sensorimotor network, occipital network, and psychomotor speed within three months after focal surgical injury in pediatric patients with intracranial space-occupying lesions
Source: BMC Pediatr. 2022 Jun 1;22:321. doi: 10.1186/s12887-022-03348-5 (PMC9158303; doi:10.1186/s12887-022-03348-5)
Supplement: Supplementary file 2 — Additional file 2: [file 12887_2022_3348_MOESM2_ESM.docx]

Supplementary Materials:

The data is from the paper “An open resource for transdiagnostic research in pediatric mental health and learning disorders” by Alexander et al.

Some important MRI parameters:

Machine: Siemens 3T Prisma;

fMRI: TR=800ms; TE=30ms; Volumes= 375 (scanning time= 5.1min);

T1 MPRAGE: TR=2500ms; TE=3.15ms; Slices= 224

We processed the data with the same pre-processing steps and used two-sample T-test (Surgery group minus control group) with gaussian random-field theory (GRF) correction (voxel p-value <0.001, cluster p-value <0.05, two-tailed) for Reho and seed-based analysis of the six ROIs. Two-sample T-test was also performed for local efficiency, global efficiency and characteristic shortest path length of graph theory analysis.

We show the results by table and figures as follows. The anatomical lables were selected from the AAL3 atlas, which is same as our original text.

1.Reho results:

(1) Positive activation

Supplementary Table 1 Reho changes of positive activation

|  | Peak Labels | Peak Intensity | MNI coordinates  (X Y Z) |
| --- | --- | --- | --- |
| Cluster 1 | Precuneus(R) | 7.03 | 9 -54 36 |
| Cluster 2 | Precuneus(R) | 7.00 | 28 23 33 |
| Cluster 3 | Temporal_Inf(R) | 5.52 | 51 -24 -27 |

(2) Negative activation:

Supplementary Table 2 Reho changes of negative activation

|  | Peak Labels | Peak Intensity | MNI coordinates  (X Y Z) |
| --- | --- | --- | --- |
| Cluster 1 | Occipital_Mid(L) | -6.29 | -36 -90 3 |
| Cluster 2 | Lingual(L) | -7.37 | -24 -69 -6 |
| Cluster 3 | Occipital_Mid(R) | -5.38 | 33 -87 12 |
| Cluster 4 | Lingual(R) | -6.37 | 30 -48 -6 |
| Cluster 5 | Cuneus(L) | -6.14 | 0 -93 21 |

Supplementary Figure 1 Reho changes


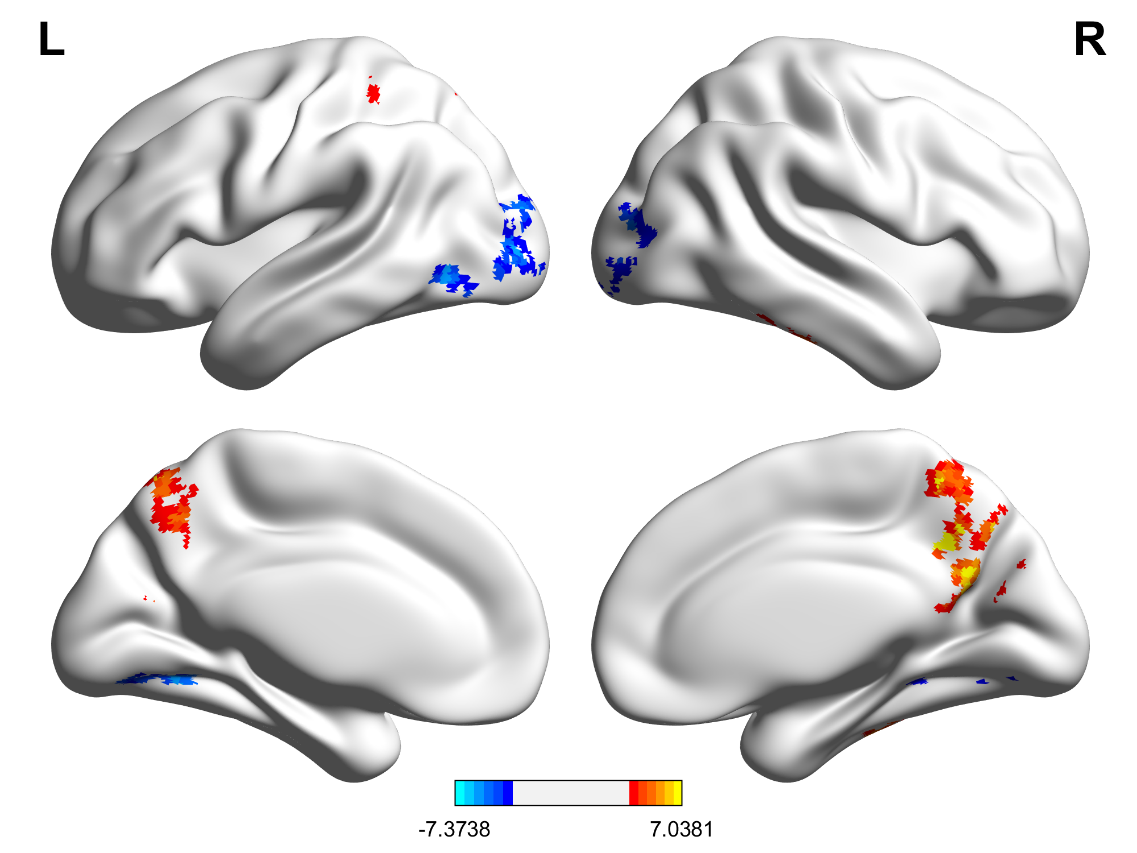


Supplementary Figure 1 shows postoperative changes in Reho compared to the control group, with most of the positive and negative activation areas within the sensorimotor network and occipital network according to anatomical location except cluster 3 in the Table 1.

2. Seed-based analysis

(1) ROI 52 (-54,-9,23) ( Postcentral_L)

Supplementary Table3 Seed-based analysis results of ROI 52

|  | Peak Labels | Peak Intensity | MNI coordinates  (X Y Z) |
| --- | --- | --- | --- |
| Cluster 1 | Postcentral(L) | -8.09 | -51 -6 27 |
| Cluster 2 | Frontal_Mid_2(R) | -6.98 | 57 -9 51 |

Supplementary Figure 2 Seed-based analysis results of ROI 52


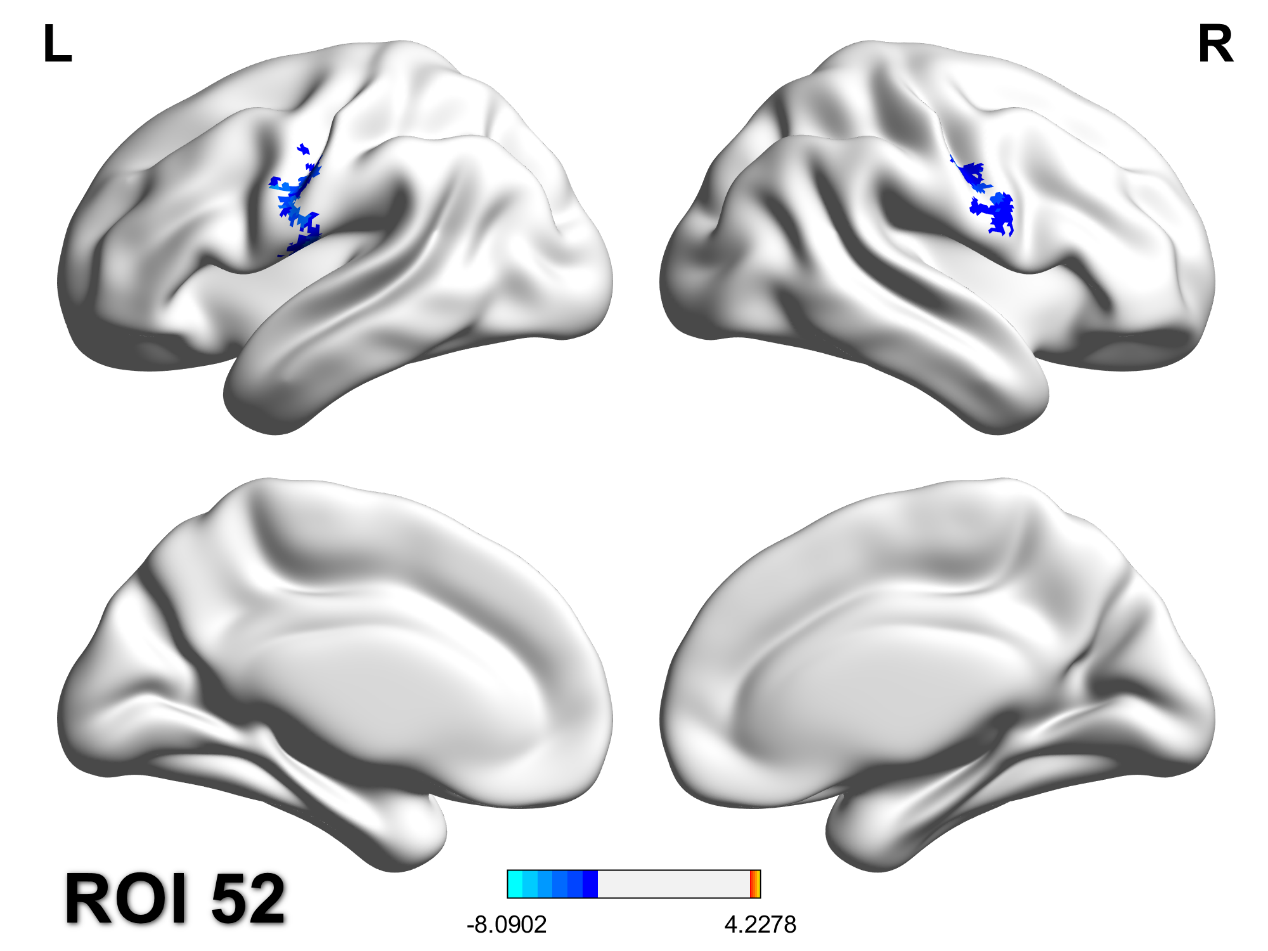


Supplementary Figure 2 shows postoperative changes compared to the control group. Cluster 1 is in the sensorimotor network and occipital network, and cluster 2 partially according to anatomy.

(2) ROI 66 (-55,-22,38) (Parietal_Inf_L)

Supplementary Table 4 Seed-based analysis results of ROI 66

|  | Peak Labels | Peak Intensity | MNI coordinates  (X Y Z) |
| --- | --- | --- | --- |
| Cluster 1 | Postcentral(L) | -6.99 | -48 -24 45 |
| Cluster 2 | Postcentral(R) | -6.89 | 33 -42 72 |
| Cluster 3 | Cingulate_Mid(R) | -5.41 | 3 -12 36 |
| Cluster 4 | Cerebellum_8(L) | -5.15 | -24 -51 -54 |
| Cluster 5 | Precentral(R) | -5.63 | 54 -15 45 |
| Cluster 6 | Cerebellum_8(R) | -5.50 | 27 -48 -51 |
| Cluster 7 | Parietal_Sup(L) | -4.89 | -27 -45 72 |

Supplementary Figure 3(a) Seed-based analysis results of ROI 66 (without cerebellum)


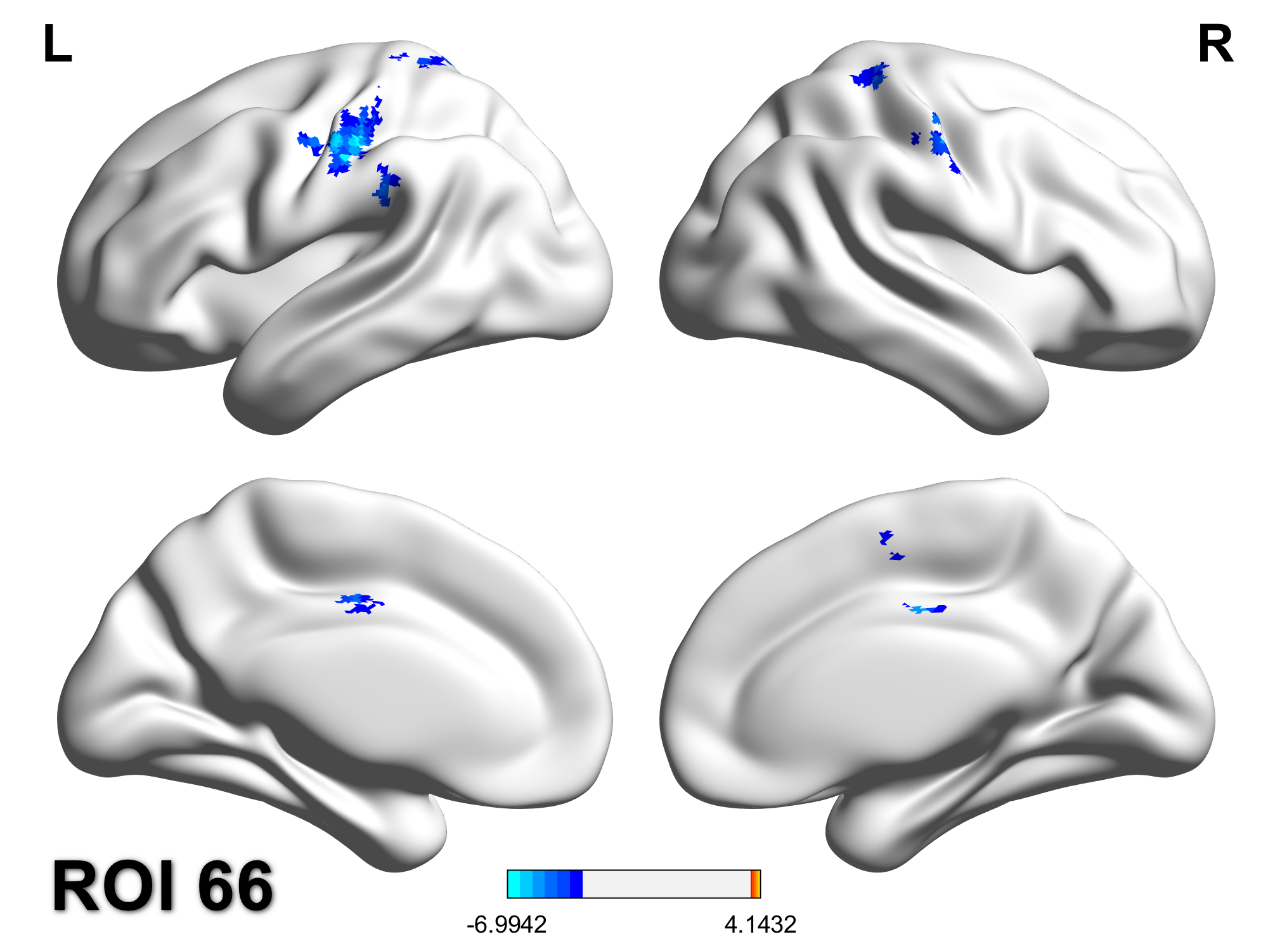


Supplementary Figure 3(b) Seed-based analysis results of ROI 66 (only show the activation of cerebellum)


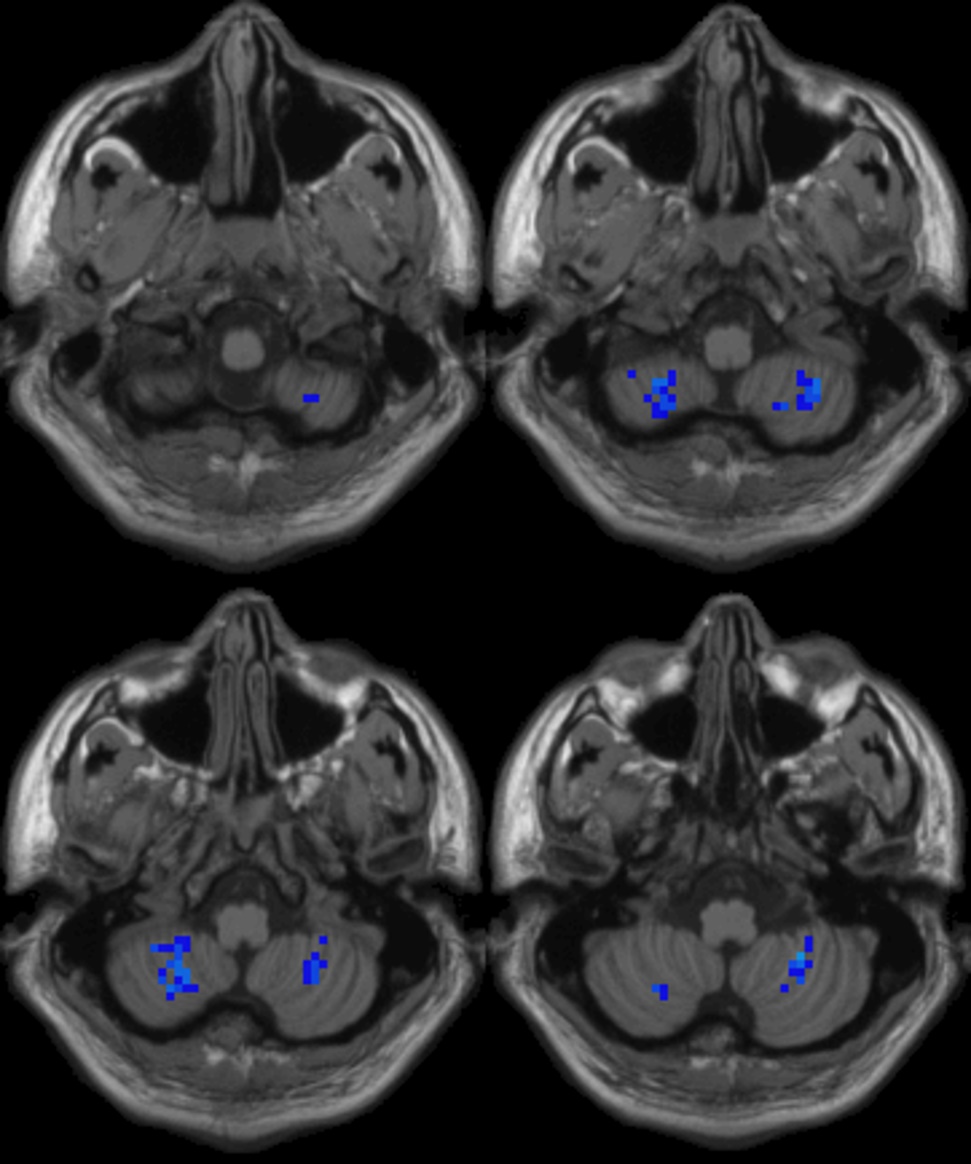


Supplementary Figure 3 (a) shows the cerebrum results and Supplementary Figure 3 (b) shows the cerebellum results. Supplementary Figure 3 (a) and (b) share the same colorbar. The cluster 1, 2, and 5 belong to sensorimotor network and occipital network partially or totally based on anatomy. The others are not.

(3) ROI 82 (-41,-37,16) (Temporal_Sup_L)

Supplementary Table 5 Seed-based analysis results of ROI 82

|  | Peak Labels | Peak Intensity | MNI coordinates  (X Y Z) |
| --- | --- | --- | --- |
| Cluster 1 | Precentral(R) | -4.79 | 24 -15 75 |
| Cluster 2 | Postcentral(L) | -4.73 | -54 -9 15 |

Supplementary Figure 4 Seed-based analysis results of ROI 82


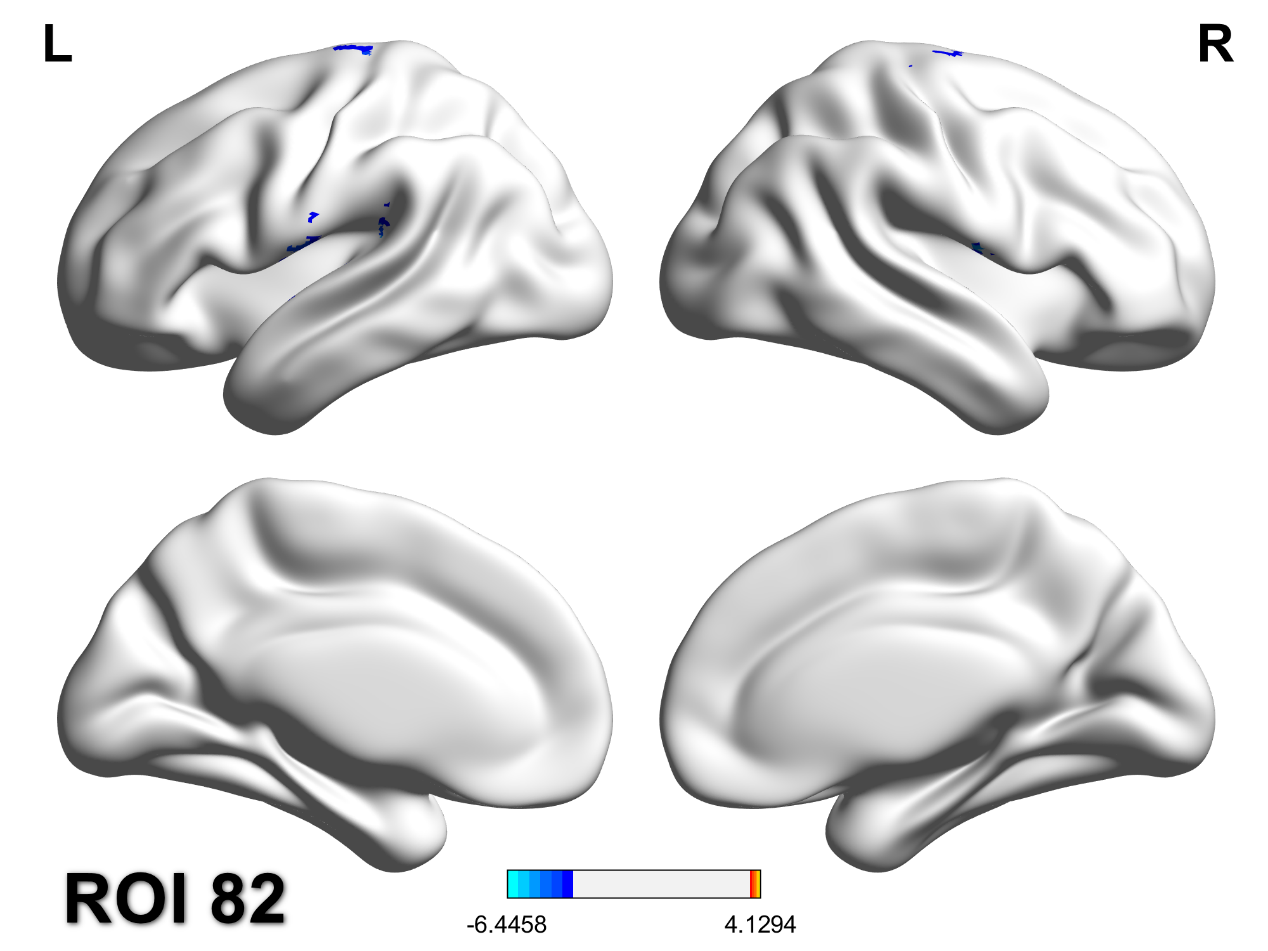


Supplementary Figure 4 shows postoperative changes compared to the control group. Both of the two cluster located in sensorimotor and occipital network anatomically.

(4) ROI 139 (29,-73,29) (Occipital_Mid_R)

Supplementary Table 6 Seed-based analysis results of ROI 139

|  | Peak Labels | Peak Intensity | MNI coordinates  (X Y Z) |
| --- | --- | --- | --- |
| Cluster 1 | Fusiform(L) | -8.38 | -36 -63 -12 |
| Cluster 2 | Occipital_Mid(R) | -8.55 | 30 -72 30 |
| Cluster 3 | Occipital_Sup(L) | -5.94 | -21 -84 39 |
| Cluster 4 | None | -5.46 | 36 -66 -6 |
| Cluster 5 | Cuneus(L) | -5.60 | 0 -84 39 |

Supplementary Figure 5 Seed-based analysis results of ROI 139


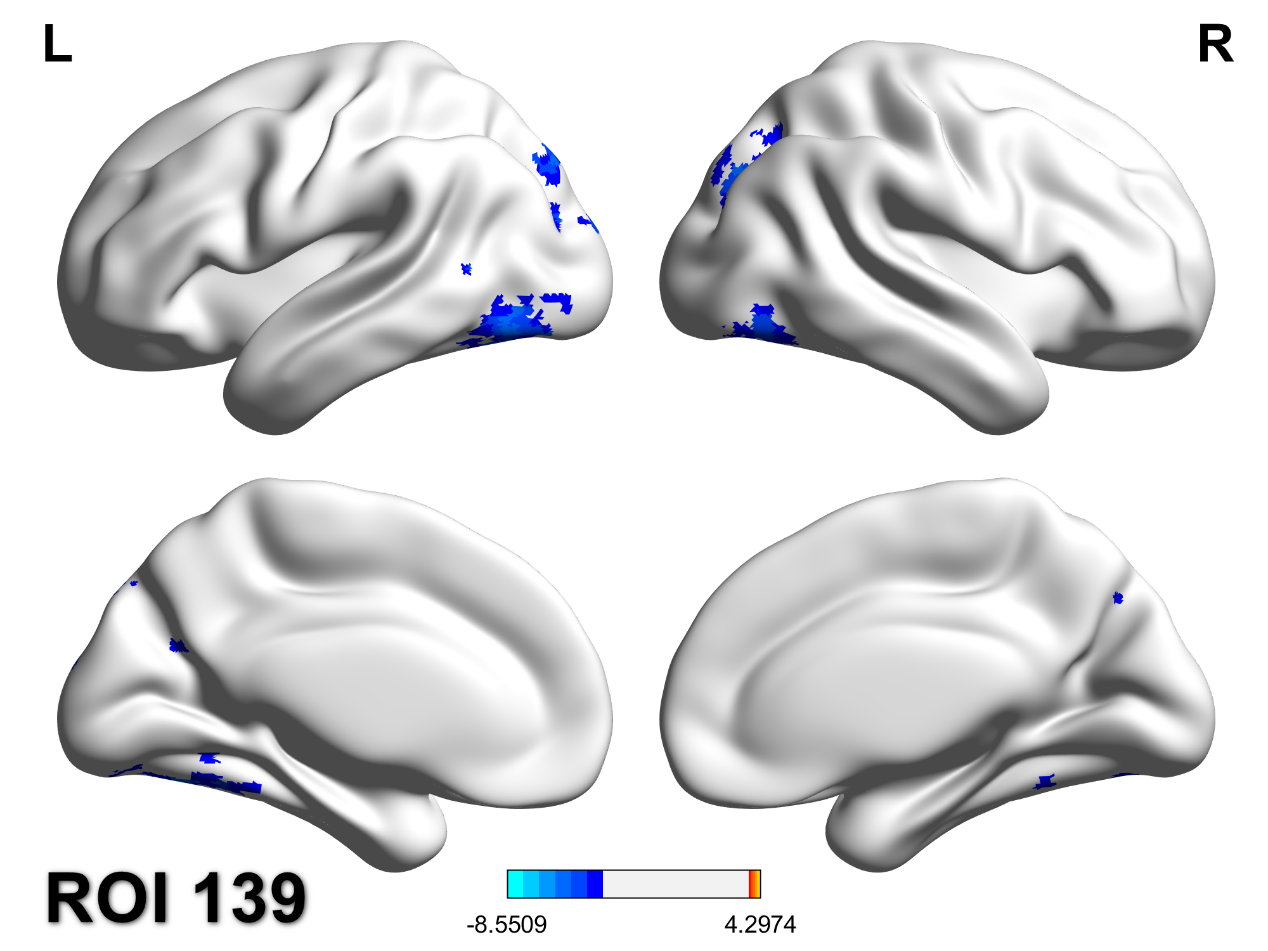


Supplementary Figure 5 shows postoperative changes compared to the control group. According to anatomy, all the five clusters are in sensorimotor and occipital networks partially or totally.

(5) ROI 145 (-16,-76,33) (Cuneus_L)

Supplementary Table 7 Seed-based analysis results of ROI 145

|  | Peak Labels | Peak Intensity | MNI coordinates  (X Y Z) |
| --- | --- | --- | --- |
| Cluster 1 | Precuneus(R) | -8.54 | 12 -75 48 |
| Cluster 2 | Parietal_Sup(L) | -6.68 | -15 -78 48 |
| Cluster 3 | Precentral(R) | -4.93 | 45 6 33 |

Supplementary Figure 6 Seed-based analysis results of ROI 145


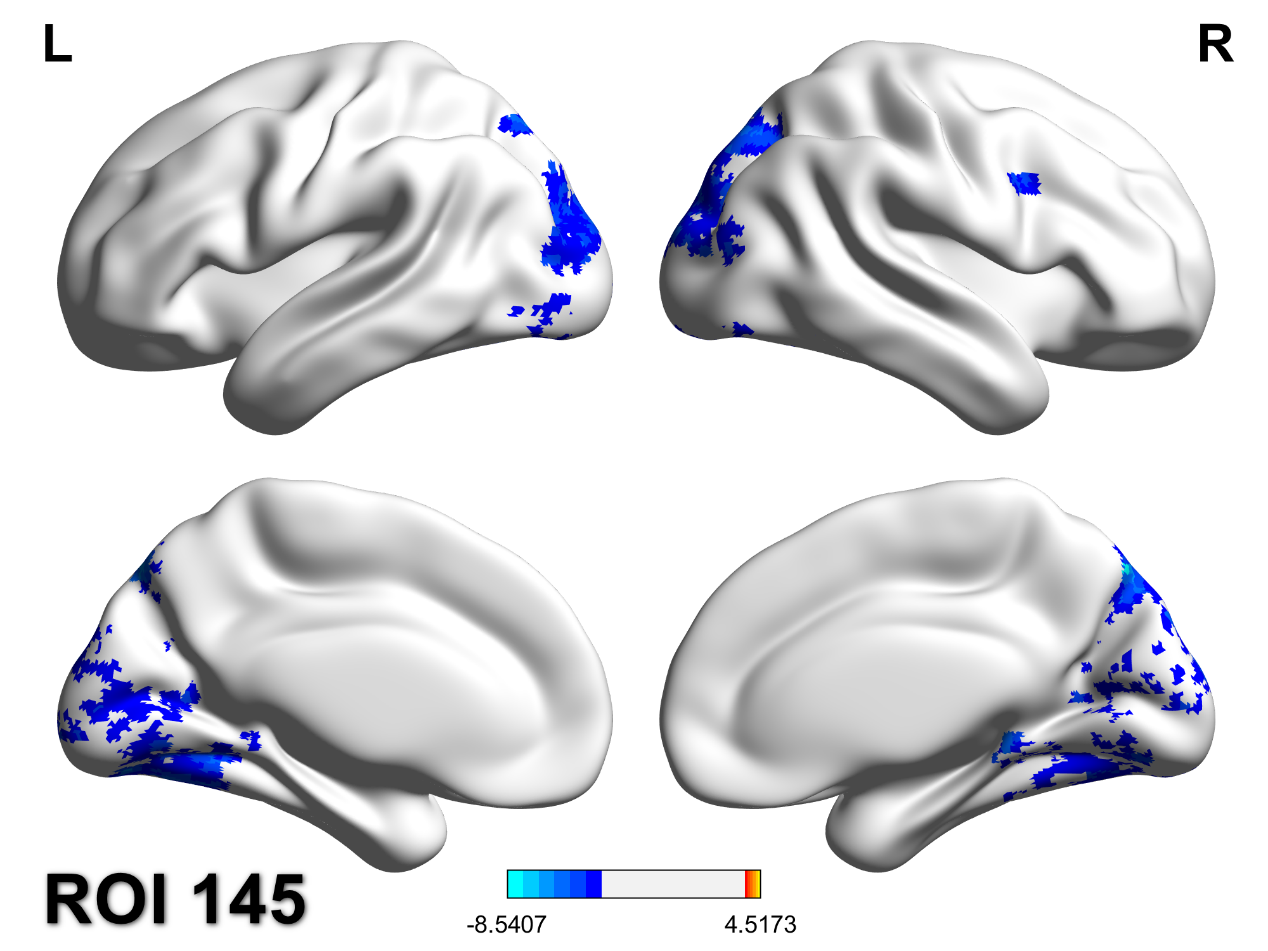


Supplementary Figure 6 shows postoperative changes compared to the control group. Cluster 2 are near sensorimotor and occipital network but not involved in them anatomically. Cluster 1 and 3 are in sensorimotor and occipital networks based on anatomy.

(6) ROI 152 (-5,-80,9) (Calcarine_L)

Supplementary Table 8 Seed-based analysis results of ROI 152

|  | Peak Labels | Peak Intensity | MNI coordinates  (X Y Z) |
| --- | --- | --- | --- |
| Cluster 1 | Calcarine(L) | -8.99 | -9 -72 12 |

Supplementary Figure 7 Seed-based analysis results of ROI 152


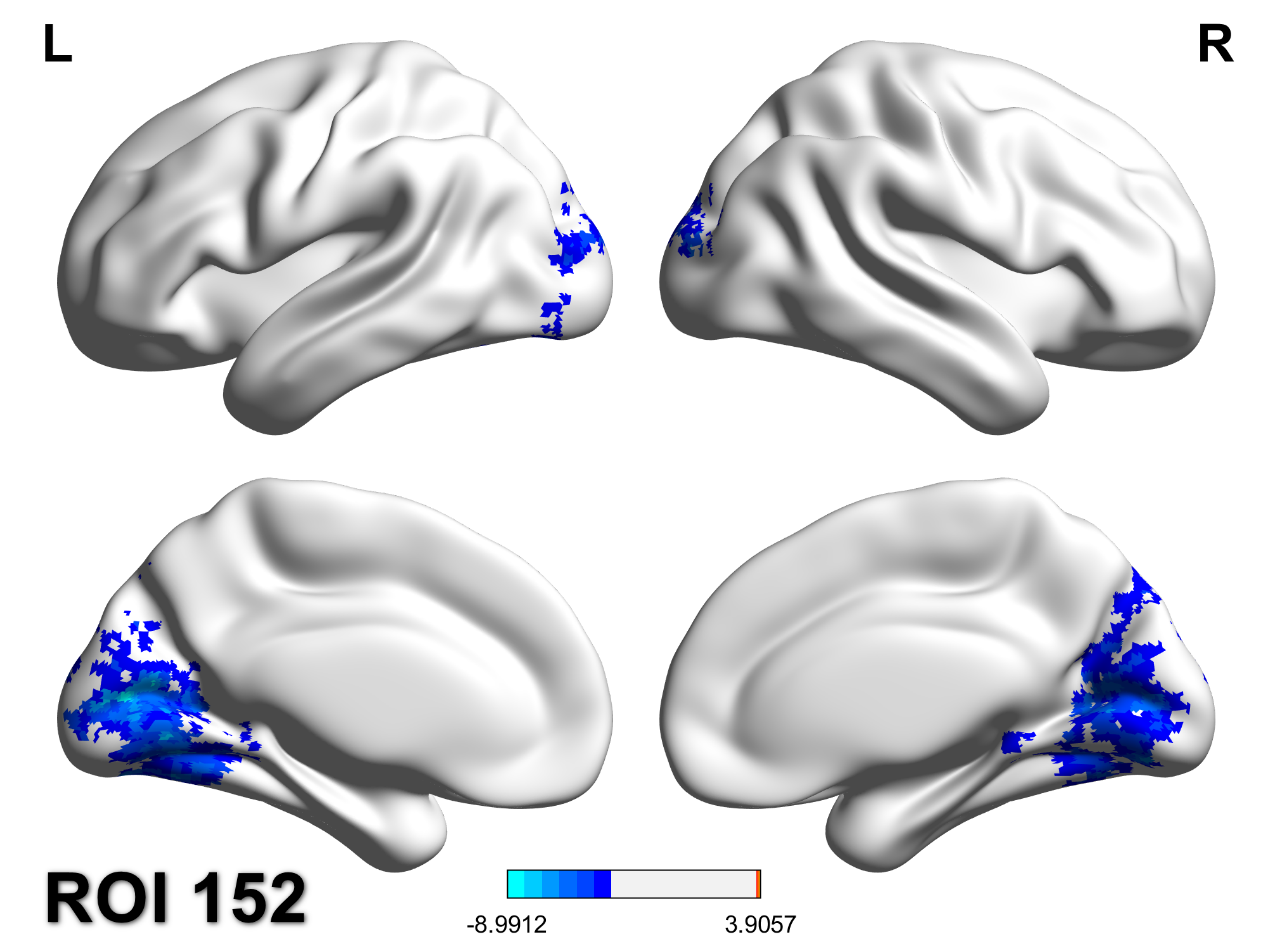


Supplementary Figure 7 shows postoperative changes compared to the control group. The cluster belongs to occipital network anatomically.

3. Graph theory analysis:

Supplementary Table 9 Graph theory analysis results of local efficiency, global efficiency and characteristic shortest path length

|  | t-value | p-value |
| --- | --- | --- |
| local efficiency | -1.33 | 0.19 |
| global efficiency | -1.10 | 0.28 |
| characteristic shortest path length | 0.39 | 0.70 |

Based on these results, we found positive activation in Reho results, which may be due to changes in the brain networks of patients with intracranial space-occupying lesion. Negative activation, similar to our results, is largely located within the sensorimotor network. In the seed-based analysis, the results were partly the same as ours, which could reflect the damage of the sensorimotor network and occipital network. It also includes other networks with reduced activation. The three indicators in graph theory analysis did not reach statistical significance, and there may be another compensation mechanism.

The above results are partially similar to our results, and the differences may be due to the changes in preoperative brain network of patients with intracranial space-occupying lesions, different data sources, or other mechanisms involved, which need to be further studied.
